# Supplementary material for: Quantitative Analysis of the Human Semen Phosphorometabolome by 31P-NMR
Source: Int J Mol Sci. 2024 Jan 30;25(3):1682. doi: 10.3390/ijms25031682 (PMC10855173; doi:10.3390/ijms25031682)
Supplement: Supplementary file 1 [file ijms-25-01682-s001.zip › ijms-2818842-supplementary.pdf]

International Journal of
Molecular Sciences

MDPI

Supplementary Materials

Table S1: Metabolites assigned in the ejaculates, seminal plasma and spermatozoa from human samples. Note: TCA= tricarboxylic acid cycle; PPP= pentose phosphate pathway.

NMR	Metabolite	Abbreviation	Metabolism
^{1}H	Choline	CHO	Lipid
	Citrate	CIT	TCA
	Tyrosine	Tyr	Amino acid
	Glutamine	Gln	Amino acid
	Phenylalanine	Phe	Amino acid
	Lactate	LAC	Glycolisis
^{31}P	Glycerophosphoinositol	GPI	Lipid
	Glycerophosphoethanolamine	GPE	Lipid
	Glycerophospho(N-biotin)ethanolamine	GP(N-biotin)E	Lipid
	Glycerophospho(monomethyl)ethanolamine	GP(monomethyl)E	Lipid
	Glycerophospho(dimethyl)ethanolamine	GP(dimethyl)E	Lipid
	Phosphocholine	PCh	Lipid
	Glycerophosphocholine	GPC	Lipid
	Glucose 1-phosphate	G1P	Glycogenolysis
	Glucose 6-phosphate	G6P	Glycolisis
	Sedoheptulose 7-phosphate and Fructose 1,6-bisphosphate	S7P&FBP	PPP/Glycolisis
	6-Phosphogluconic acid	6PG	PPP
	Acetyl phosphate	AcP	Other
	Xylulose 5-phosphate	X5P	PPP
	Nicotinamide adenine dinucleotide phosphate reduced form	NADPH	Energy
	Nucleotide monophosphate	NMP	Nucleotide
	Cyclic nucleotide monophosphate	NMPc	Nucleotide
	Phosphodiester unknown 1	PDE1	Unknown
	Phosphodiester unknown 2	PDE2	Unknown
	Phosphodiester unknown 3	PDE3	Unknown
	Phosphodiester unknown 4	PDE4	Unknown

Table S2: Complete list of information of each metabolite concentration in the seminal plasma (SP) samples.

	Seminal plasma samples (nmol/μL)																		
	Donors with normozoospermia										Donors with asthenozoospermia								
Metabolite	Mean	SEM	STD	1	2	3	4	5	6	7	Mean	SEM	STD	1	2	3	4	5	6
CHO *	25.05	2.38	6.30	18.57	27.23	30.16	30.15	27.21	28.18	13.88	16.00	2.57	6.30	25.89	18.06	15.66	17.64	11.04	7.68
Citrate	18.48	0.72	1.91	18.86	19.45	17.50	15.69	18.12	21.87	17.90	19.38	0.92	2.25	23.40	19.81	18.22	16.92	18.19	19.70
Tyr *	5.34	0.43	1.14	7.05	5.45	4.78	5.25	5.92	5.64	3.32	3.64	0.57	1.39	5.11	1.78	3.45	5.17	3.91	2.40
LAC	3.72	1.07	2.82	2.89	2.70	1.81	2.41	3.09	3.13	10.03	3.06	0.31	0.77	2.72	2.10	3.68	2.46	4.12	3.30
Gln	2.36	0.05	0.14	2.37	2.38	2.33	2.40	2.08	2.51	2.48	2.43	0.04	0.09	2.45	2.44	2.38	2.59	2.37	2.33
Phe*	2.28	0.20	0.52	2.83	2.35	2.18	2.38	2.45	2.56	1.20	1.53	0.27	0.67	2.15	0.65	1.28	2.41	1.62	1.07
GPC	9.85	1.83	4.84	12.12	7.52	6.26	8.03	7.35	7.66	19.99	8.93	1.30	3.19	9.64	9.71	3.65	7.64	13.37	9.55
GPI	1.82	0.17	0.45	1.39	2.71	1.40	1.89	1.64	1.80	1.91	1.20	0.25	0.61	1.85	0.86	0.44	1.91	1.39	0.74
NMP	1.10	0.26	0.68	0.19	1.51	1.69	1.27	1.51	1.46	0.05	1.10	0.21	0.51	1.46	1.71	0.90	1.47	0.56	0.51
GP(dimethyl)E	0.67	0.05	0.13	0.59	0.76	0.80	0.47	0.59	0.83	0.63	0.59	0.04	0.10	0.61	0.58	0.66	0.38	0.64	0.64
GPE	0.49	0.17	0.44	0.59	0.36	0.27	0.21	0.24	0.33	1.45	0.48	0.13	0.32	0.26	0.63	0.23	0.20	1.01	0.55
G1P	0.20	0.05	0.13	0.29	0.13	0.13	0.12	0.12	0.15	0.45	0.39	0.11	0.26	0.11	0.34	0.39	0.12	0.76	0.59
G6P	0.37	0.07	0.19	0.08	0.35	0.32	0.32	0.35	0.43	0.71	0.18	0.06	0.14	0.31	0.32	0.02	0.28	0.08	0.06
Pcho *	0.05	0.01	0.02	0.03	0.04	0.04	0.04	0.04	0.04	0.09	0.53	0.44	1.07	0.05	2.70	0.25	0.04	0.07	0.06
S7P&FBP	0.17	0.04	0.10	0.35	0.17	0.12	0.13	0.16	0.21	0.03	0.09	0.03	0.07	0.19	0.05	0.04	0.17	0.04	0.04
PDE2	0.08	0.01	0.02	0.09	0.09	0.08	0.05	0.09	0.07	0.10	0.07	0.02	0.04	0.07	0.09	0.05	0.05	0.14	0.04

**6PG**	0.06	0.01	0.03	0.04	0.07	0.06	0.05	0.07	0.11	0.03	0.06	0.01	0.03	0.06	0.08	0.05	0.06	0.02	0.10
**NADPH**	0.054	0.015	0.041	0.09	0.03	0.03	0.02	0.03	0.05	0.13	0.047	0.010	0.024	0.03	0.08	0.02	0.03	0.07	0.05
**PDE4**	0.054	0.012	0.030	0.08	0.03	0.05	0.03	0.05	0.03	0.11	0.043	0.007	0.018	0.05	0.04	0.01	0.05	0.05	0.06
**GP(N-biotin)E**	0.050	0.007	0.018	0.03	0.05	0.03	0.04	0.06	0.06	0.08	0.035	0.008	0.021	0.04	0.03	0.01	0.02	0.04	0.07
**PDE1**	0.046	0.003	0.008	0.05	0.04	0.04	0.04	0.05	0.06	0.04	0.038	0.004	0.010	0.04	0.04	0.04	0.02	0.05	0.04
**AcP**	0.026	0.004	0.010	0.01	0.03	0.04	0.02	0.03	0.03	0.02	0.030	0.007	0.018	0.02	0.04	0.01	0.02	0.06	0.03
**NMPc**	0.029	0.005	0.012	0.01	0.04	0.04	0.03	0.04	0.02	0.02	0.023	0.004	0.010	0.04	0.02	0.01	0.03	0.02	0.02
**X5P**	0.020	0.000	0.000	0.02	0.02	0.02	0.02	0.02	0.02	0.02	0.020	0.000	0.000	0.02	0.02	0.02	0.02	0.02	0.02
**GP(monomethyl)E**	0.026	0.006	0.017	0.01	0.03	0.02	0.03	0.04	0.05	0.00	0.012	0.006	0.015	0.03	0.00	0.00	0.03	0.01	0.00
**PDE3**	0.010	0.000	0.000	0.01	0.01	0.01	0.01	0.01	0.01	0.01	0.010	0.000	0.000	0.01	0.01	0.01	0.01	0.01	0.01
**Semen volume (mL)**	3.071	0.561	1.484	2.00	3.00	4.00	6.00	2.00	2.50	2.00	3.17	0.38	0.93	3.50	4.00	1.50	4.00	3.00	3.00

Table S3: Complete list of information of each metabolite concentration in unprocessed semen samples. Note: ND= no data.

	Whole semen samples (nmol/µL)																		
	Donors with normozoospermia										Donors with asthenozoospermia								
**Metabolite**	**Mean**	**SEM**	**STD**	**1**	**2**	**3**	**4**	**5**	**6**	**7**	**Mean**	**SEM**	**STD**	**1**	**2**	**3**	**4**	**5**	**6**
CHO *	27.07	2.94	7.21	ND	34.60	29.37	22.92	31.17	29.79	14.55	16.41	3.39	7.59	28.12	ND	14.90	19.13	10.08	9.82
Citrate	26.20	1.91	4.67	ND	23.86	32.17	23.91	31.37	20.23	25.63	22.09	1.45	3.25	20.82	ND	21.02	18.24	23.51	26.85
Tyr *	5.70	0.57	1.39	ND	6.22	5.94	5.05	7.85	5.48	3.63	3.53	0.46	1.04	4.94	ND	2.91	4.32	2.92	2.57
Gln	4.11	0.51	1.25	ND	4.95	5.17	4.14	5.17	2.25	2.95	3.06	0.58	1.29	1.32	ND	4.16	2.06	3.63	4.12
LAC	4.03	1.34	3.28	ND	2.36	2.09	4.38	2.22	2.63	10.50	3.15	0.49	1.11	1.67	ND	3.29	2.47	3.84	4.47
Phe *	2.21	0.22	0.54	ND	2.82	1.88	2.14	2.79	2.21	1.42	1.57	0.26	0.58	2.45	ND	1.19	1.86	1.25	1.10
GPC	9.06	1.18	2.88	ND	8.13	6.65	10.34	7.95	6.97	14.32	9.09	1.01	2.27	10.74	ND	7.52	8.41	12.11	6.68
GP(dimethyl)E	0.70	0.02	0.05	ND	0.67	0.73	0.68	0.65	0.79	0.69	0.69	0.03	0.07	0.73	ND	0.71	0.57	0.70	0.72
GPE	0.69	0.09	0.22	ND	0.78	0.73	0.66	0.67	0.32	0.98	0.64	0.07	0.15	0.61	ND	0.49	0.66	0.88	0.54
NMP	0.57	0.10	0.25	ND	0.80	0.74	0.61	0.67	0.48	0.10	0.78	0.15	0.35	0.75	ND	1.32	0.87	0.52	0.45
GPI	0.43	0.14	0.34	ND	0.22	0.31	0.60	0.22	0.18	1.06	0.71	0.24	0.53	0.22	ND	0.70	0.16	1.27	1.22
G6P	0.59	0.07	0.16	ND	0.65	0.74	0.47	0.79	0.38	0.53	0.38	0.17	0.39	0.70	ND	0.13	0.90	0.13	0.06
G1P	0.09	0.01	0.04	ND	0.11	0.10	0.02	0.10	0.11	0.11	0.46	0.25	0.55	0.09	ND	0.34	0.10	1.42	0.33
PCho	0.13	0.02	0.04	ND	0.15	0.18	0.13	0.14	0.14	0.05	0.19	0.07	0.16	0.12	ND	0.47	0.14	0.10	0.13
S7P&FBP	0.11	0.01	0.04	ND	0.13	0.13	0.11	0.13	0.13	0.04	0.09	0.02	0.05	0.13	ND	0.12	0.13	0.04	0.04
PDE1	0.09	0.01	0.03	ND	0.12	0.12	0.09	0.07	0.05	0.08	0.08	0.00	0.01	0.08	ND	0.09	0.08	0.09	0.08
6PG	0.07	0.01	0.02	ND	0.07	0.07	0.08	0.07	0.07	0.03	0.07	0.01	0.03	0.07	ND	0.11	0.07	0.02	0.09

PDE2	0.06	0.01	0.02	ND	0.05	0.05	0.09	0.05	0.04	0.09	0.08	0.01	0.03	0.07	ND	0.10	0.09	0.10	0.04
NADPH	0.05	0.01	0.01	ND	0.05	0.05	0.05	0.04	0.05	0.08	0.04	0.01	0.02	0.04	ND	0.07	0.03	0.06	0.02
PDE4	0.05	0.01	0.01	ND	0.04	0.04	0.04	0.06	0.04	0.07	0.04	0.01	0.01	0.04	ND	0.03	0.03	0.04	0.06
NMPc	0.04	0.01	0.02	ND	0.04	0.06	0.03	0.04	0.04	0.01	0.04	0.01	0.02	0.06	ND	0.02	0.04	0.01	0.06
GP(N-biotin)E	0.05	0.00	0.01	ND	0.04	0.05	0.04	0.06	0.05	0.05	0.04	0.01	0.02	0.05	ND	0.02	0.04	0.03	0.06
PDE3	0.03	0.00	0.01	ND	0.04	0.02	0.03	0.03	0.03	0.03	0.03	0.00	0.00	0.03	ND	0.03	0.03	0.03	0.03
GP(monomethyl)E	0.04	0.01	0.02	ND	0.05	0.05	0.03	0.05	0.05	0.01	0.03	0.01	0.02	0.05	ND	0.01	0.05	0.01	0.01
AcP	0.03	0.00	0.01	ND	0.03	0.02	0.03	0.04	0.02	0.02	0.04	0.01	0.01	0.03	ND	0.03	0.03	0.06	0.04
X5P	0.02	0.00	0.00	ND	0.02	0.02	0.02	0.02	0.02	0.02	0.02	0.00	0.00	0.02	ND	0.02	0.02	0.02	0.02

Table S4: Complete list of information of each metabolite concentration in the spermatozoa samples (CELL) analyzed from donors with normozoospermia.

		Spermatozoa samples (nmol/Millions of cells)						
		Donors with normozoospermia						
**NMR**	**Metabolite**	**1**	**2**	**3**	**4**	**5**	**6**	**7**
1H	**Citrate**	37.02	13.44	11.78	20.97	18.18	31.18	16.59
1H	**CHO**	15.18	12.22	13.67	20.63	12.79	27.20	12.47
1H	**Tyr**	5.10	2.34	3.06	2.80	2.99	7.48	2.98
1H	**LAC**	7.25	1.07	1.17	1.65	2.88	3.05	9.23
1H	**Gln**	6.21	1.69	2.45	3.21	2.80	4.63	2.57
1H	**Phe**	1.95	1.16	1.89	1.95	1.79	3.53	1.27
31P	**GPC**	6.50	2.74	2.97	4.14	4.53	9.88	4.20
31P	**NMP**	1.01	1.40	1.59	1.67	1.82	4.28	0.16
31P	**S7P&FBP**	0.03	0.53	0.56	0.89	0.54	1.28	0.00
31P	**GPE**	0.29	0.30	0.24	0.37	0.31	0.48	0.24
31P	**G1P**	0.13	0.34	0.13	0.43	0.29	0.95	0.10
31P	**GPI**	0.49	0.19	0.16	0.28	0.28	0.28	0.41
31P	**G6P**	0.07	0.15	0.15	0.24	0.11	0.33	0.04
31P	**PCho**	0.16	0.06	0.05	0.07	0.07	0.13	0.03
31P	**PDE4**	0.03	0.07	0.07	0.09	0.05	0.15	0.03
31P	**PDE3**	0.13	0.05	0.05	0.07	0.07	0.10	0.06
31P	**PDE2**	0.03	0.03	0.05	0.09	0.07	0.10	0.03
31P	**GP(dimethyl)E**	0.10	0.05	0.03	0.06	0.05	0.08	0.04
31P	**GP(N-biotin)E**	0.00	0.03	0.05	0.04	0.05	0.10	0.01
31P	**X5P**	0.07	0.02	0.02	0.04	0.03	0.05	0.03
31P	**PDE1**	0.07	0.02	0.02	0.04	0.03	0.05	0.03
31P	**6PG**	0.03	0.03	0.02	0.04	0.03	0.05	0.00
31P	**NMPc**	0.00	0.02	0.02	0.06	0.02	0.05	0.00
31P	**AcP**	0.00	0.02	0.02	0.04	0.02	0.03	0.01
31P	**GP(monomethyl)E**	0.03	0.01	0.02	0.02	0.02	0.03	0.00
31P	**NADPH**	0.03	0.01	0.01	0.02	0.02	0.03	0.01

Table S5: Overrepresented pathways using the phosphorylated metabolites identified and quantificated by 31P -NMR in the ejaculate, seminal plasma and spermatozoa from human.

**Metabolic pathway**	***p*-value**	**Metabolite**
Pentose phosphate pathway	2.29×10^{-5}	X5P ǀ 6PG ǀ G6P ǀ S7P
Signal transduction/ cell signaling	4.10×10^{-5}	NMP ǀ NMPc
Metabolic pathways	5.63×10^{-5}	NADPH ǀ NMP ǀ G6P<br>G1P ǀ Pch ǀ AcP ǀ X5P ǀ 6PG ǀ FBP ǀ GPC<br>GPI ǀGPEǀ S7P
Glycerophospholipid metabolism	9.89×10^{-5}	Pch ǀ GPC ǀ GPI ǀ GPE ǀ GP(N-biotin)E<br>GP(dimethyl)E ǀ GP(monomethyl)E
Purine metabolism	1.43×10^{-3}	NMP ǀ NMPc
Starch and sucrose metabolism	2.46×10^{-3}	G6P ǀ G1P
Glycolysis / Glycogenolysis	1.25×10^{-2}	G6P ǀ G1P
Inositol phosphate metabolism	1.94×10^{-2}	G6P ǀ GPI
Pentose and glucuronate interconversions	3.44×10^{-2}	G1P ǀ X5P
Biosynthesis of secondary metabolites	3.36×10^{-1}	NMP ǀ G6P ǀ G1P ǀ X5P ǀ 6PG ǀ S7P

Table S6: Complete list of the relative amounts of each metabolite in spermatozoa (CELL) and seminal plasma (SP) from each donor.

	Donors with normozoospermia																			
	Relative amounts of metabolites in spermaozoa (%)										Relative amounts of metabolites in seminal plasma (%)									
Metabolite	Mean	SEM	STD	1	2	3	4	5	6	7	Mean	SEM	STD	1	2	3	4	5	6	7
Citrate **	41.39	1.99	5.26	50.92	42.09	34.62	40.94	43.90	40.46	36.77	32.42	1.32	3.49	35.88	32.66	29.78	26.92	30.78	34.23	36.67
CHO **	33.35	2.75	7.27	20.88	38.28	40.18	40.28	30.87	35.30	27.64	43.27	3.22	8.52	35.32	45.72	51.33	51.73	46.22	44.11	28.44
Tyr	7.48	0.54	1.43	7.02	7.34	9.00	5.47	7.22	9.70	6.61	9.34	0.78	2.06	13.41	9.15	8.13	9.01	10.06	8.83	6.80
LAC	7.34	2.38	6.31	9.97	3.34	3.44	3.23	6.94	3.96	20.46	6.85	2.30	6.09	5.50	4.53	3.08	4.14	5.25	4.90	20.55
Gln **	6.54	0.41	1.08	8.54	5.31	7.20	6.27	6.75	6.00	5.70	4.16	0.19	0.50	4.51	4.00	3.97	4.12	3.53	3.93	5.08
Phe	3.91	0.38	1.01	2.68	3.64	5.56	3.81	4.32	4.57	2.82	3.96	0.32	0.86	5.38	3.95	3.71	4.08	4.16	4.01	2.46
GPC	56.52	4.69	12.41	70.67	44.95	47.58	47.65	54.07	53.74	76.96	62.81	3.67	9.70	75.42	53.75	54.67	62.64	58.85	57.08	77.27
NMP *	18.09	3.08	8.16	10.95	23.05	25.46	19.23	21.71	23.27	2.98	8.54	2.11	5.59	1.18	10.79	14.76	9.91	12.09	10.88	0.19
S7P&FBP	5.95	1.57	4.15	0.35	8.76	8.92	10.26	6.40	6.94	0.00	1.20	0.24	0.62	2.18	1.22	1.05	1.01	1.28	1.56	0.12
GPE	3.84	0.30	0.79	3.18	4.95	3.90	4.27	3.68	2.59	4.34	2.89	0.51	1.36	3.67	2.57	2.36	1.64	1.92	2.46	5.60
G1P **	3.49	0.65	1.73	1.41	5.52	2.04	4.91	3.49	5.17	1.90	1.23	0.14	0.38	1.80	0.93	1.14	0.94	0.96	1.12	1.74
GPI	3.79	0.76	2.02	5.30	3.05	2.60	3.21	3.29	1.50	7.59	12.70	1.50	3.96	8.65	19.37	12.23	14.74	13.13	13.41	7.38
G6P	1.76	0.31	0.83	0.71	2.48	2.42	2.78	1.36	1.77	0.81	2.43	0.33	0.89	0.50	2.50	2.79	2.50	2.80	3.20	2.74
Pcho **	0.90	0.15	0.40	1.77	0.95	0.74	0.85	0.78	0.68	0.54	0.30	0.02	0.06	0.19	0.29	0.35	0.31	0.32	0.30	0.35
PDE4 **	0.80	0.12	0.32	0.35	1.14	1.12	1.07	0.58	0.82	0.54	0.35	0.05	0.12	0.50	0.21	0.44	0.23	0.40	0.22	0.43
PDE3 **	0.88	0.11	0.28	1.41	0.76	0.74	0.85	0.78	0.54	1.08	0.07	0.01	0.02	0.06	0.07	0.09	0.08	0.08	0.07	0.04
PDE2	0.66	0.09	0.23	0.35	0.57	0.74	1.07	0.78	0.54	0.54	0.56	0.05	0.14	0.56	0.64	0.70	0.39	0.72	0.52	0.39

**GP(dimethyl)E ****	0.69	0.08	0.21	1.06	0.76	0.56	0.64	0.58	0.41	0.81	4.73	0.60	1.59	3.67	5.43	6.99	3.67	4.72	6.18	2.44
**GP(N-biotin)E**	0.45	0.09	0.25	0.00	0.57	0.74	0.43	0.58	0.54	0.27	0.34	0.04	0.10	0.19	0.36	0.26	0.31	0.48	0.45	0.31
**X5P ****	0.44	0.05	0.14	0.71	0.38	0.37	0.43	0.39	0.27	0.54	0.14	0.01	0.03	0.12	0.14	0.17	0.16	0.16	0.15	0.08
**PDE1**	0.44	0.05	0.14	0.71	0.38	0.37	0.43	0.39	0.27	0.54	0.32	0.04	0.09	0.31	0.29	0.35	0.31	0.40	0.45	0.15
**6PG**	0.34	0.07	0.18	0.35	0.57	0.37	0.43	0.39	0.27	0.00	0.45	0.09	0.23	0.25	0.50	0.52	0.39	0.56	0.82	0.12
**NMPc**	0.27	0.09	0.23	0.00	0.38	0.37	0.64	0.19	0.27	0.00	0.21	0.04	0.12	0.06	0.29	0.35	0.23	0.32	0.15	0.08
**AcP**	0.25	0.06	0.15	0.00	0.38	0.37	0.43	0.19	0.14	0.27	0.19	0.04	0.10	0.06	0.21	0.35	0.16	0.24	0.22	0.08
**NADPH**	0.22	0.03	0.07	0.35	0.19	0.19	0.21	0.19	0.14	0.27	0.33	0.06	0.15	0.56	0.21	0.26	0.16	0.24	0.37	0.50
**GP(monomethyl)E**	0.21	0.05	0.13	0.35	0.19	0.37	0.21	0.19	0.14	0.00	0.20	0.05	0.13	0.06	0.21	0.17	0.23	0.32	0.37	0.00
